# Association between high cost user status and end-of-life care in hospitalized patients: A national cohort study of patients who die in hospital

**DOI:** 10.1177/02692163211002045

**Published:** 2021-03-30

**Authors:** Kieran L Quinn, Amy T Hsu, Christopher Meaney, Danial Qureshi, Peter Tanuseputro, Hsien Seow, Colleen Webber, Rob Fowler, James Downar, Russell Goldman, Raphael Chan, Kimberlyn McGrail, Sarina R Isenberg

**Affiliations:** 1 Department of Medicine, University of Toronto, Toronto, ON, Canada; 2 ICES, Toronto and Ottawa, ON, Canada; 3 Institute of Health Policy, Management and Evaluation, University of Toronto, Toronto, ON, Canada; 4 Department of Medicine, Sinai Health System, Toronto, ON, Canada; 5 Clinical Epidemiology Program, Ottawa Hospital Research Institute, Ottawa, ON, Canada; 6 School of Epidemiology, Public Health and Preventive Medicine, University of Ottawa, Ottawa, ON, Canada; 7 Bruyère Research Institute, Ottawa, ON, Canada; 8 Department of Family and Community Medicine, University of Toronto, Toronto, ON, Canada; 9 Division of Palliative Care, Department of Medicine, University of Ottawa, Ottawa, ON, Canada; 10 Department of Oncology, McMaster University, Hamilton, ON, Canada; 11 Tory Trauma Program, Sunnybrook Hospital, Interdepartmental Division of Critical Care Medicine, Department of Medicine, University of Toronto, Toronto, Ontario; 12 Temmy Latner Centre for Palliative Care and Lunenfeld Tanenbaum Research Institute, Sinai Health System, Toronto, ON, Canada; 13 Centre for Health Services and Policy Research, School of Population and Public Health, The University of British Columbia, Vancouver, BC, Canada; 14 Department of Medicine, University of Ottawa, ON, Canada

**Keywords:** High cost users, delivery of healthcare, end-of-life, palliative care

## Abstract

**Background:**

Studies comparing end-of-life care between patients who are high cost users of the healthcare system compared to those who are not are lacking.

**Aim:**

The objective of this study was to describe and measure the association between high cost user status and several health services outcomes for all adults in Canada who died in acute care, compared to non-high cost users and those without prior healthcare use.

**Settings and participants:**

We used administrative data for all adults who died in hospital in Canada between 2011 and 2015 to measure the odds of admission to the intensive care unit (ICU), receipt of invasive interventions, major surgery, and receipt of palliative care during the hospitalization in which the patient died. High cost users were defined as those in the top 10% of acute healthcare costs in the year prior to a person’s hospitalization in which they died.

**Results:**

Among 252,648 people who died in hospital, 25,264 were high cost users (10%), 112,506 were non-high cost users (44.5%) and 114,878 had no prior acute care use (45.5%). After adjustment for age and sex, high cost user status was associated with a 14% increased odds of receiving an invasive intervention, a 15% increased odds of having major surgery, and an 8% lower odds of receiving palliative care compared to non-high cost users, but opposite when compared to patients without prior healthcare use.

**Conclusions:**

Many patients receive aggressive elements of end-of-life care during the hospitalization in which they die and a substantial number do not receive palliative care. Understanding how this care differs between those who were previously high- and non-high cost users may provide an opportunity to improve end of life care for whom better care planning and provision ought to be an equal priority.

**What is already known about this topic?**

- Patients in the top 10% of healthcare expenditure (“high cost users”) account for 50% of annual healthcare costs and approximately 10%–13% of these costs were devoted to the care of patients in their last year of life.
- Few studies compare the use of acute care services at the end-of-life according to patients’ prior healthcare use, so the question remains, “how much more likely are the highest cost users getting potentially low-value services?”

**What this paper adds**

- Fifteen to twenty five percent of all patients received more aggressive elements of end-of-life care during the hospitalization in which they died.
- High cost user status was associated with a 14% increased odds of receiving an invasive intervention (adjusted odds ratio (aOR) 1.14, 95% CI 1.09–1.20), a 15% increased odds of having major surgery (aOR 1.15, 95% CI 1.10–1.20), and an 8% lower odds of receiving palliative care (aOR 0.92, 95% CI 0.89–0.95).

**Implications for practice**

- Understanding how this care differs between those who were previously high- and non-high cost users may provide an opportunity to improve end of life care for whom better care planning and provision ought to be an equal priority.

## Introduction

The care of high-cost, seriously ill patients at or near the end of life presents a vexing challenge to health care systems, requiring a focus on the delivery of high-quality care in line with their expressed preferences. Prior work demonstrated that the top 10% of healthcare cost users (“high cost users”) account for 50% of annual healthcare costs and approximately 10%–13% of these costs were devoted to the care of patients in their last year of life.^1–3^ Almost half of the costs incurred near end-of-life were related to acute care.^1,4,5^

In general, end-of-life care that is delivered in the acute care setting is expensive, may be of limited benefit, and is associated with poor quality of life.^1,6–13^ When asked, the majority of patients report wanting to have their treatment preferences in writing.^14^ They also prefer to focus on treatments that provide comfort over survival at the end of life.^15,16^ In spite of these findings, more than half of these patients are admitted to intensive care units at the end of life.^17^ The delivery of specific clinical interventions such as admission to the intensive care unit (ICU), mechanical ventilation and major surgery at the very end of life may therefore be viewed as of low- or uncertain-value for many patients.^18^

High-value care is care that reflects patient preferences, is focused on outcomes, and is cost-efficient in achieving those outcomes.^19^ In the context of end-of-life care, there is evidence that this approach improves the dying experience and controls costs.^20–22^ Healthcare systems are increasingly focused on delivering high-value end-of-life care as the prevalence of chronic disease and its related healthcare expenditure continue to rise. Incorporating palliative care as a component of inpatient care for patients nearing the end of life may improve value by enhancing outcomes such as quality of life and reducing symptom burden. A key function of palliative care teams is the reorientation of healthcare provision to ensure optimal care in line with patients’ and their family’s needs. Although not its intended purpose, palliative care may simultaneously reduce healthcare use and its associated costs in patients opting for less aggressive and generally more expensive care.^23–29^ Yet one third of patients who die in hospital do not access palliative care.^30^ One approach to improving value at the end-of-life may be to focus efforts on the specific subgroup of high cost patients at a unique juncture in time—to prevent potentially unwanted aggressive treatment, such as during the hospitalization in which they die.^4,18,20,31^ Understanding how end of life care differs between those who were previously high- and non-high cost users may provide an opportunity that allows clinicians to focus discussions potential low-value care to ensure care at the end of life is aligned with patient preferences.

The question then remains, “how much more likely are the highest cost users getting potentially low-value services?” Few studies compare the use of acute care services at the end-of-life according to patients’ prior healthcare use. The objective of this study was to describe and measure the association between high cost user status and several health services outcomes for all adults in Canada who died in acute care, compared to non-high cost users and those without prior healthcare use. High cost user status was defined as being in the top 10% of acute care costs in the 12 months prior to hospitalization in which the patient died. Outcomes included the odds of being admitted to an intensive care unit (ICU), of receiving invasive interventions (defined as mechanical ventilation, resuscitation, newly initiated dialysis, percutaneous coronary intervention, bronchoscopy, use of feeding tubes, or receipt of blood transfusion), major surgery and palliative care.

## Methods

### Study design, setting, and data sources

We conducted a national retrospective cohort study in Canada, using health administrative data from the Discharge Abstract Database (DAD) between April 1st, 2011 and March 31st, 2015. The DAD is a national database that contains patient-level data for all acute care institutions in Canada (excluding the province of Quebec). All residents of Canada have universal access to hospital care and medically necessary physicians’ services. Canada is a high-income nation with the world’s 10th highest gross domestic product.

### Study cohort

Our cohort included all Canadian adults who died in hospital between April 1st, 2012 and March 31st, 2015. We excluded people with a hospitalization length of stay ⩾6 months due to the high costs incurred from long hospital admissions because they would bias the results as de facto high cost users. It is not typical for most patients to remain this long in hospital, and these patients are often waiting for care settings that can address their specific care needs related to their inability to function independently at home. We also excluded those who were <18, those at extremes of age who were >105 years old as these traditionally tend to represent outliers in the data (and comprised less than 0.02% of the eligible patients), or who were non-Canadian residents.

### Patient characteristics

We measured demographic and clinical variables based on data from their prior hospitalizations including age, sex, rural location of residence, comorbidities and chronic conditions,^32^ hospital frailty risk score,^33^ and their use of acute health care services in the 12 months prior to the admission date of the hospitalization in which the patient died.

### Costing prior acute care use and defining high user status

We used the distribution of the costs of patients’ hospitalizations in the 12 months prior to the hospitalization in which the patient died to inform the threshold for defining high user status (Supplemental Tables S1 and S2). We intentionally used costs over other frequency measures of healthcare use because costs encompass all acute care received and are therefore a more comprehensive surrogate measure of total acute care use.

The total costs of a patient’s prior acute care usage over the 12-months preceding the hospitalization in which the patient died were obtained using validated costing methods.^34–36^ Briefly, each acute care hospital admission is associated with a resource intensity weight (RIW). RIWs measure the intensity of resource use associated with different medical, diagnostic and surgical procedures, adjusted for patient characteristics and characteristics of the institution such as case-mix adjustments. These RIWs are multiplied by a cost-of-standard-hospital-stay (CSHS) value measured at the provincial level. We adjusted per-admission costs using the Statistics Canada Health Care Consumer Price Index (CPI), using 2014 as a base-year.^37^

We created three groups of patients according to their CPI adjusted per-patient costs associated with their use of acute care in the prior 12 months: high cost users, non-high cost users, and those without prior acute care use. High user status was defined as being in the top decile of acute healthcare costs in the year prior to the hospitalization in which the patient died. We used this threshold based on its prior use in the literature and the distribution of costs among our cohort (Supplemental Figure S1).^2^

### Outcomes

The main outcomes were individual measures of aggressive end-of-life care, palliative care and a patient’s approach to care, defined as having a palliative intent. We specifically measured admission to the ICU, receipt of invasive interventions (defined as mechanical ventilation, resuscitation, newly initiated dialysis, percutaneous coronary intervention, bronchoscopy, use of feeding tubes, or receipt of blood transfusion), major surgery, or palliative care. The specific components of invasive interventions were selected based on a review of the literature and the expert opinions of specialists in internal medicine, palliative care and geriatrics.^7^ These interventions are common, costly, and some are associated with discomfort. They are therefore of uncertain benefit as they do not necessarily improve the dying experience at the end of life.

Major surgical procedures were captured based on a defined set of procedure codes and were categorized according to anatomy: abdominal, cardiac, retroperitoneal, thoracic, and vascular (Supplemental Table S3).^38–40^ We identified the delivery of palliative care using two separate methods. First, we identified a binary measure of the delivery of palliative care comprised of no palliative care vs. palliative care, which could consist of one of three scenarios: (1) when a patient’s most responsible diagnosis (MRDx) or other diagnoses code was “palliative care—Z51.5,” (2) their most responsible physician (MRP) was “palliative care” or they were seen by a palliative care physician during their admission, or (3) a healthcare provider other than a physician delivered palliative care (e.g. nurse or nurse practitioner, social work, or spiritual care clinician).

We then categorized a patient’s care as having a palliative intent according to the approach taken during the hospitalization in which the patient died. These approaches are coded by trained chart abstractors at the Canadian Institutes for Health Information (CIHI). A patient’s approach to care was classified as delivered with “palliative intent likely” when MRDx and/or MRP were palliative care, “palliative intent unlikely” when a patient had a diagnosis other than MRDx listed as palliative care, and “no palliative intent” when a patient had neither a diagnosis nor MRP listed as palliative care.

### Statistical analysis

The association between high user status versus non-high user status or having no prior healthcare use and the odds of receiving aggressive end-of-life care, palliative care or having an approach to care with a palliative intent during the hospitalization in which the patient died was measured using a Generalized Estimating Equation (GEE) framework to model the adjusted impact of high user status on our outcomes. All models considered were adjusted for age and sex and used a compound symmetric working correlation structure, where patient level outcomes were nested within a particular treatment facility. Dichotomous outcomes were modeled using a logistic GEE approach (admission to ICU, receipt of invasive interventions, major surgery and palliative care); whereas, categorical outcomes were modeled using a multinomial GEE approach (approach to care). As the presence of a high burden of chronic disease is recognized as one the six phenotypes of high cost users,^4^ we intentionally did not adjust for comorbidity because it may be directly related to high user status and may result in multicollinearity in our regression analysis.

All analyses were performed using SAS version 9.4 (SAS Institute, Cary, North Carolina).

## Results

### Baseline characteristics

**Figure 1.**
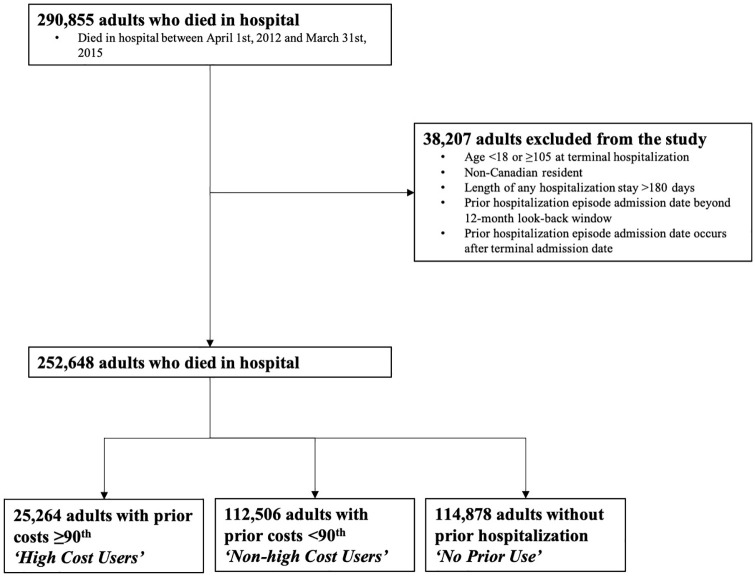
Flow diagram for the creation of the study sample. All adults who died in hospital in Canada between April 1st, 2011 and March 31st, 2015 were assessed for inclusion in the study. Patients were stratified into high cost users, non-high cost users and those without prior use based on their acute care use in the 12 months preceding the hospitalization in which they die.

There were 290,855 patients who died in hospital during the study period. Of these, 38,207 people were excluded from the study. The final cohort consisted of 252,648 people, 25,264 who were high cost users (10%), 112,506 who were non-high cost users (44.5%) and 114,878 who had no prior acute care use (45.5%) (Figure 1).

High cost users were typically younger (median age 74 vs 78 years), had higher hospital frailty risk scores (mean score 3.3 vs 1.8), higher acute healthcare use in the prior 12 months (median number of hospitalizations 3 vs 1) and more prior exposure to inpatient palliative care (17.8% vs 10.7%) than non-high cost users (Table 1). A higher proportion of high cost users compared to non-high cost users also had chronic terminal noncancer illnesses such as heart failure (34.2% vs 20.3%), chronic obstructive pulmonary disease (25.9 vs 17.9%), and chronic kidney disease (35.2% vs 15.6%).

**Table 1.** Baseline characteristics in patients who died in hospital by user group in Canada between 2011 and 2015.

	Healthcare user group (*n* = 252,648)		
	Non-high cost user^a^ (*n* = 112,506)	High cost user^a^ (*n* = 25,264)	No prior use user^a^ (*n* = 114,878)
Age (years), median (IRQ)	78 (67–86)	74 (63–83)	80 (69–87)
Age Group (years), *n* (%)			
18–45	2804 (2.5)	1304 (5.2)	3401 (3.0)
46–55	6550 (5.8)	2132 (8.4)	5783 (5.0)
56–65	15,130 (13.4)	4416 (17.5)	12,870 (11.2)
66–70	11,084 (9.9)	2931 (11.6)	9560 (8.3)
71–75	13,132 (11.7)	3120 (12.3)	11,946 (10.4)
76–80	15,919 (14.1)	3467 (13.7)	15,476 (13.4)
81–85	19,098 (17.0)	3664 (14.5)	19,628 (17.1)
86–90	17,102 (15.2)	2757 (10.9)	19,656 (17.1)
91–95	9217 (8.2)	1197 (4.7)	12,347 (10.8)
⩾96	2470 (2.2)	276 (1.1)	4211 (3.7)
Female sex, *n* (%)	52,962 (47.1)	11,423 (45.2)	54,996 (47.9)
Rurality, *n* (%)	23,209 (20.7)	5210 (20.7)	21,902 (19.1)
Chronic conditions, *n* (%)			
Cancer	41,008 (36.4)	9914 (39.2)	−^b^
Hypertension	38,423 (34.2)	12,807 (50.7)	−^b^
Diabetes	29,809 (26.5)	9491 (37.6)	−^b^
Congestive heart failure	22,859 (20.3)	8629 (34.2)	−^b^
Chronic obstructive pulmonary disease	20,160 (17.9)	6537 (25.9)	−^b^
Chronic kidney disease	17,530 (15.6)	8903 (35.2)	−^b^
Coronary syndrome (excluding AMI)	14,594 (13.0)	5774 (22.9)	−^b^
Dementia	9028 (8.0)	2448 (9.7)	−^b^
Cardiac arrhythmia	6430 (5.7)	2910 (11.5)	−^b^
Myocardial infarction	6245 (5.6)	2726 (10.8)	−^b^
Mood, anxiety, depression and other nonpsychotic disorders	4884 (4.3)	3181 (12.6)	−^b^
Stroke (excluding transient ischemic attack)	3373 (3.0)	1518 (6.0)	−^b^
Osteoarthritis	3244 (2.9)	1240 (4.9)	−^b^
Osteoporosis	1743 (1.5)	727 (2.9)	−^b^
Hospital frailty score, *n* (%)			−^b^
Mean (SD)	1.8 (0.4-4.1)	3.3 (1.6-5.8)	−^b^
Healthcare system use 12 months prior to hospitalization in which the patient died			
No. Inpatient admissions, median (IQR)	1 (1–2)	3 (2–4)	−^b^
Hospital LOS, median (IQR)	11 (5–20)	55 (38–79)	−^b^
No. ICU admissions, *n* (%)			
0	96,827 (86.1)	14,415 (57.1)	−^b^
1	12,774 (11.4)	6077 (24.1)	−^b^
⩾2	2905 (2.5)	4772 (18.8)	−^b^
Received inpatient palliative care in the 12 months prior to hospitalization in which the patient died, *n* (%)	12,058 (10.7)	4493 (17.8)	−^b^

IQR: interquartile range; ICU: intensive care unit; LOS: length of stay.

a High cost users are defined as those in the top 10% of acute care costs based on use in the prior 12 months. Non-high cost users are those in the bottom 90% of acute care costs who had at least one acute care admission in the prior 12 months. No prior use users are those in the bottom 90% of acute care costs who had no acute care admission in the prior 12 months.

b These baseline characteristics are unavailable because they are determined from prior hospitalization data in the 12 months prior to the hospitalization in which the patient died.

Patients with no prior healthcare use in the 12 months prior to the hospitalization in which they died were slightly older (mean age 80 years) and a similar proportion were female (47.9%) and lived in rural areas (19.1%), as compared to other the other healthcare user groups.

### High user status and aggressive end-of-life care

A slightly larger proportion of high cost users had at least one admission to the ICU compared to non-high cost users (27.5% vs 25.2%). However, after adjustment for age and sex, high user status was not associated with ICU admission (unadjusted odds ratio (OR) 1.18, 95% CI 1.11–1.25; adjusted OR (aOR) 1.03, 95% CI 0.98–1.08). A larger proportion of patients without prior healthcare use had at least one admission to the ICU compared to high cost users (35.3%). After adjustment for age and sex, high user status was associated with a lower odds of ICU admission compared to having no prior healthcare use (OR 0.72, 95% CI 0.67–0.77; aOR 0.57, 95% CI 0.54–0.61).

Among high cost users, 25.7% received at least one invasive intervention, compared with 21.0% of non-high cost users. High user status was associated with a 14% increased odds of receiving an invasive intervention (OR 1.34, 95% CI 1.27–1.42; aOR 1.14, 95% CI 1.09–1.20) compared to non-high cost users. A larger proportion of patients without prior healthcare received at least one invasive intervention, compared to high cost users (31.0%). After adjustment for age and sex, high user status was associated with a lower odds of receiving an invasive intervention compared to having no prior healthcare use (OR 0.78, 95% CI 0.72–0.85; aOR 0.61, 95% CI 0.57–0.64).

A larger proportion of high cost users received major surgery during the hospitalization in which the patient died than non-high cost users, which was associated with a higher odds of having major surgery (20.3% vs 16.2%, respectively; OR 1.35, 95% CI 1.27–1.42; aOR 1.15, 95% CI 1.10–1.20). A larger proportion of patients without prior healthcare received major surgery compared to high cost users (21.2%). After adjustment for age and sex, high user status was associated with a lower odds of having major surgery compared to having no prior healthcare use (OR 0.79, 95% CI 0.75–0.84; aOR 0.79, 95% CI 0.75–0.84).

**Figure 2.**
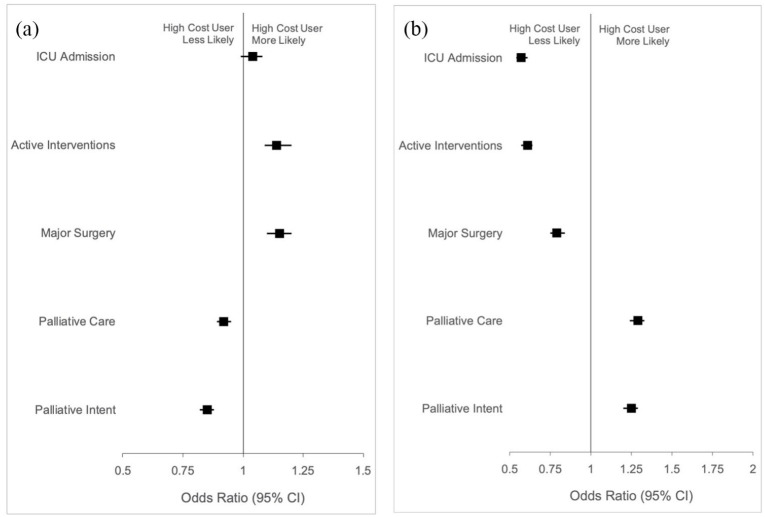
Association between user cost status and aggressive elements of end-of-life care or palliative care during the hospitalization in which the patient died, among adults who died in hospital in Canada between 2011 and 2015. Models compared (a) high to non-high cost users and (b) high cost users to those with no prior use and were adjusted for age and sex. ICU: Intensive care unit.

A similar proportion of high- and non-high cost users received palliative care during the hospitalization in which they died (64.1% vs 65.4%). However, after adjustment for age and sex, high user status was associated with a lower odds of receiving any form of palliative care compared to non-high cost users (OR 0.94, 95% CI 0.92–0.97; aOR 0.92, 95% CI 0.89–0.95). Among high cost users, 24.6% had an approach to care with “palliative intent likely,” 5.9% with “palliative intent unlikely” and 69.5% with “no palliative intent.” Among non-high cost users, 27.5% had an approach to care with “palliative intent likely,” 6.1% with “palliative intent unlikely” and 66.4% with “no palliative intent.” After adjustment for age and sex, high cost user status was associated with a lower odds of having a palliative approach to care relative to other approaches, compared to non-high cost users (OR 0.92, 95% CI 0.90–0.95; aOR 0.92, 95% CI 0.89–0.95) (Table 2, Figure 2). A smaller proportion of patients without prior healthcare use received palliative care compared to high cost users (58.2%). After adjustment for age and sex, high user status was associated with a higher odds of receiving any form of palliative care, compared to having no prior healthcare use (OR 1.29, 95% CI 1.24–1.34; aOR 1.29, 95% CI 1.24–1.33). Among those without prior healthcare use, 19.9% had an approach to care with “palliative intent likely,” 34.8% with “palliative intent unlikely” and 45.3% with “no palliative intent.” After adjustment for age and sex, high user status was associated with a higher odds of having a palliative approach to care relative to other approaches, compared to having no prior use (OR 1.25, 95% CI 1.21–1.30; aOR 1.25, 95% CI 1.20–1.29).

**Table 2.** Delivery of end-of-life care during the hospitalization in which the patient died by user group among adults who die in hospital in Canada between 2011 and 2015.

	Healthcare user group (*n* = 252,648)		
	Non-high cost user^a^ (*n* = 112,506)	High cost user^a^ (*n* = 25,264)	No prior use user^a^ (*n* = 114,878)
ICU admission, *n* (%)	28,348 (25.2)	6958 (27.5)	40,521 (35.3)
Invasive interventions, *n* (%)	23,667 (21.0)	6502 (25.7)	35,577 (31.0)
Mechanical ventilation	18,387 (16.3)	4609 (18.2)	29,771 (25.9)
Cardiopulmonary resuscitation	6552 (4.4)	1528 (4.6)	7641 (6.7)
Feeding tube	3392 (2.3)	895 (2.7)	3074 (2.7)
Defibrillation	2323 (1.6)	538 (1.6)	1942 (1.7)
Bronchoscopy	1569 (1.1)	440 (1.3)	1817 (1.6)
Percutaneous coronary intervention	792 (0.5)	124 (0.4)	3518 (3.1)
Transfusion	116 (0.1)	92 (0.3)	52 (0.05)
Major surgery	18,237 (16.2)	5125 (20.3)	24,326 (21.2)
Received palliative care, *n* (%)	73,558 (65.4)	16,187 (64.1)	66,900 (58.2)
Approach to care, *n* (%)			
Palliative intent likely	30,947 (27.5)	6226 (24.6)	22,899 (19.9)
Palliative intent unlikely	6839 (6.1)	1484 (5.9)	39,924 (34.8)
No palliative intent	74,720 (66.4)	17,554 (69.5)	52,055 (45.3)

IQR: interquartile range; ICU: intensive care unit; COPD: chronic obstructive pulmonary disease.

a High cost users are defined as those in the top 10% of acute care costs based on prior years use. Non-high cost users are those in the bottom 90% of acute care costs who had at least one acute care admission in the prior 12 months. No prior use users are those in the bottom 90% of acute care costs who had no acute care admission in the prior 12 months.

Overall, the association between high cost user status and both clinical and cost outcomes were stable over the study’s timeframe.

## Discussion

### Main findings

Our cohort study of 252,648 adult patients who died in hospital found that people who were previously in the highest decile of acute healthcare expenditure (“high cost users”) were more likely to receive an invasive intervention or major surgery, and less likely to receive palliative care or be treated with a palliative intent than non-high cost users during the hospitalization in which they died. No association was observed between high cost users and admission to ICU, compared to non-high cost users. We found the opposite results when comparing high cost users to patients without prior healthcare use. More broadly, one third of the entire study sample did not receive palliative care during the hospitalization in which they died.

Our findings demonstrate that patients who were previously high cost users of acute care generally were more likely to receive aggressive elements of care at the end of life compared to non-high cost users but less likely than patients without prior healthcare use. It is unclear whether what we defined as aggressive end-of-life care is a reflection of the underlying treatment preferences of patients or their substitute decision makers, which were not directly measured in this study, or of clinical inertia that sometimes lead to care interventions that worsen quality of life and do not improve survival outcomes. We included blood transfusion as part of care deemed to be invasive because it requires the use of an intravenous and because blood is a finite resource. Although transfusion at the end of life is controversial, it may be useful to improve survival and provide symptom relief in some patients.^41^ Regardless of healthcare user status, clinicians should always attempt to clarify treatment preferences to ensure the care their patients receive is appropriately aligned with them. A careful discussion that weighs the risks and benefits of different approaches to end-of-life care may identify patients who wish to decline more aggressive treatments but who may have otherwise received them.^20,42^

Similar to prior studies, we found that high cost users accounted for comparable annual acute healthcare costs and costs at the end of life.^2,4^ A three-year longitudinal population-based study of high cost users who were in the top 5% of healthcare spending in Ontario, Canada concluded that improving healthcare value may be achieved by focusing efforts on high cost users to avoid potentially low-value care.^2^ A recent analysis of wasteful spending in the United States estimated the costs of low-value care at $12.8 billion to $28.6 billion USD. Specifically, the authors suggested that scaling effective interventions that use strategies to reduce overuse, encourage shared decision-making to reduce unnecessary procedures and expand hospice and palliative care access, may help achieve most of these savings.^18^ Recent data reports that one third of patients who die in hospital do not access palliative care.^30^ Specific disease management programs may also provide benefit, given that high cost users often have a high burden of chronic disease.^5^

### Limitations

Our study has several limitations. The retrospective nature of our study limits our understanding of patients’ preferences for specific types of care interventions and how care decisions were made with their clinicians. It is not possible to establish whether low-value care is being provided without knowing patients’ preferences. We suspect that a substantial proportion of the care delivered in our study was misaligned with patient preferences because the majority of patients prefer comfort focused care over life-prolonging treatments at the end of life and prior work has demonstrated significant discordance between preferences and treatments received.^15,43^ However, there are instances (such as in certain types of cancer for example) whereby the benefits of more aggressive treatments or ICU care is uncertain near the end of life and therefore may be appropriate, especially given the heterogeneity in patient preferences for different types of care near the end of life.^44–48^ The observational nature of our study and the inability to measure a temporal relationship between the receipt of aggressive elements of end-of-life care and the timing of palliative care delivery precludes the ability to establish a causal relationship between the two. However, prior work has demonstrated lower hospital costs to be associated with the early initiation of palliative care.^49–53^ We intentionally did not adjust for co-morbidity as it is directly related to high user status and a high burden of chronic disease is recognized to define a phenotype of high cost users.^31^ Moreover, our definition of high user status was based on prior acute care costs and may exclude some high cost users whose costs are related to other care settings. Nonetheless, the objective of our study was to examine acute care use, and our cohort is derived from people who are in the last year of life where nearly half of all healthcare costs are attributable to acute care.^1^ We employed a commonly used threshold of the top decile of total prior acute care costs to define user status, but different thresholds may yield slightly different results. However, as clinicians likely use normative representations (i.e. high vs non-high) instead of strictly empirical measures of their patient’s prior healthcare use when determining user status at the time of hospital admission, the exact threshold likely becomes less important. Our costing methods represent the provincial average cost per case of inpatient care weighted for resource intensity and not actual cost at the individual patient level. Canada ranks among one of the wealthiest nations in the world and the generalizability of our findings to middle- and low-income countries is unclear. Finally, although the data in this study is older, we observed stable temporal trends in the association between high-user status and both clinical and cost outcomes, suggesting that these are salient patterns that one can expect to find in the presence of more recent data.

### What this study adds

Our study provides a window into the types of care that high cost users may be more likely to receive during the hospitalization in which they die, where clinicians may wish to focus discussions on clarifying preferences surrounding willingness to receive aggressive elements of end-of-life care, especially during situations of uncertainty. One challenge with this is approach is that physicians and patients are faced with care decisions at the time of hospital admission without knowing that it may be their last. However, most patients are hospitalized only once in their last year of life,^17^ and the continued advancement of artificial intelligence may improve our ability to identify when this is the case.^54,55^ Alternatively, having these discussions earlier, especially for older patients with complex medical illness who frequently interact with the healthcare system, may be one strategy to identify, clarify and document patient priorities for care which have been shown to reduce treatment burden and unwanted healthcare.^43,56^ Innovative strategies beyond high healthcare use may be required to identify the substantial numbers of non-high cost users in their last year of life who ultimately died in hospital. Our results may therefore highlight an opportunity to focus efforts by clinicians and systems on maximizing value to patients at the end of life, where previous efforts to improve value more broadly have yielded disappointing results.^31^

## Conclusions

Many patients receive aggressive elements of end-of-life care during the hospitalization in which they die and a substantial number do not receive palliative care. Understanding how this care differs between those who were previously high- and non-high cost users may provide an opportunity to improve end of life care for whom better care planning and provision ought to be an equal priority.

## Supplemental Material

sj-docx-1-pmj-10.1177_02692163211002045 – Supplemental material for Association between high cost user status and end-of-life care in hospitalized patients: A national cohort study of patients who die in hospital Click here for additional data file. Supplemental material, sj-docx-1-pmj-10.1177_02692163211002045 for Association between high cost user status and end-of-life care in hospitalized patients: A national cohort study of patients who die in hospital by Kieran L Quinn, Amy T Hsu, Christopher Meaney, Danial Qureshi, Peter Tanuseputro, Hsien Seow, Colleen Webber, Rob Fowler, James Downar, Russell Goldman, Raphael Chan, Kimberlyn McGrail and Sarina R Isenberg in Palliative Medicine
