# Supplementary material for: Association between high cost user status and end-of-life care in hospitalized patients: A national cohort study of patients who die in hospital
Source: Palliat Med. 2021 Mar 30;35(9):1671–81. doi: 10.1177/02692163211002045 (PMC8532234; doi:10.1177/02692163211002045)
Supplement: sj-docx-1-pmj-10.1177_02692163211002045 – Supplemental material for Association between high cost user status and end-of-life care in hospitalized patients: A national cohort study of patients who die in hospital [file sj-docx-1-pmj-10.1177_02692163211002045.docx]

**Supplementary Online Content**

**Association Between High Cost User Status and End-of-Life Care for Hospitalized Patients**

*A National Cohort Study of Canadians who Die in Hospital*

**eText 1 - Description of datasets**

**eTable 1 – Distribution of average per patient acute care costs**

**eTable 2 – Distribution of total acute care costs**

**eTable 3 – ICD-10 codes used to capture major surgery**

**eFigure 1 – Empirical cumulative distribution function (ECDF) curve of acute care costs during prior hospitalization and the hospitalization in which the patient died**

**eReferences**

**eText 1 - Description of datasets**

| **Database** | **Description** |
| --- | --- |
|  |  |
| Canadian Institute for Health Information Discharge Abstract Database (CIHI-DAD) | Contains detailed diagnostic and procedural information for all hospital admissions in Canada.  DAD records have been demonstrated to have excellent agreement (over 99%) for demographic and administrative data. Regarding diagnoses, median agreement between original DAD records and re-abstracted records for the 50 most common most responsible diagnoses was noted to be 81% (Sensitivity 82%; Specificity 82%). The corresponding median agreement for the 50 most frequently performed surgical procedures was 92% (sensitivity 95%, positive predictive value 91%).^1^ |
|  |  |

**eTable 1 – Distribution of average per patient acute care costs in patients who died in hospital by timing of hospitalization and healthcare user group in Canada between 2011 and 2015.**

|  | **All acute care users** | **No prior use^a^** | **Non-high cost users^a^** | **High cost users^a^** | **Cost Difference^d^**  **(%)** |
| --- | --- | --- | --- | --- | --- |
| **Median (IQR) cost per patient** | | | |  |  |
| Terminal hospitalization^b^ | $10,020  (4,239-24,020) | $10,336  (4,358-24,530) | $9,589  (4,062-22,363) | $11,794  (4,671-29,579) | $2,205  (23.0) |
| All admissions prior to the terminal hospitalization^c^ | $6,717  (4,148-12,004) | -- | $5,886  (3,895- 9,706) | $9,953  (5,183-25,109) | $4,067  (69.1) |
| All hospitalizations during the study period | $7,736  (4,176-16,792) | $10,336  (4,358-24,530) | $6,716  (3,938-12,620) | $10,323  (5,134-26,117) | $3,607  (53.7) |

^a^High users are defined as those in the top 10% of acute care costs based on prior years use. Non-high users are the bottom 90%.

^b^Terminal hospitalization is the hospital admission in which a person dies.

^c^Among decedents with ≥1 hospitalization record in the 12 months prior to terminal hospitalization.

^d^Cost difference between high and non-high users

**eTable 2 – Distribution of total acute care costs in patients who died in hospital by timing of hospitalization and healthcare user group in Canada between 2011 and 2015.**

|  | **All acute care users** | **No prior use^a^** | **Non-high cost users^a^** | **High cost users^a^** |  |
| --- | --- | --- | --- | --- | --- |
| **Total cost among all patients** | | | |  | **Percentage of Costs Attributed to High Users (%)** |
| Terminal hospitalization^b^ | $5,457,978,577 | $2,546,314,946 | $2,254,767,416 | $656,896,215 | 12.0 |
| All admissions prior to the terminal hospitalization^c^ | $3,267,400,046 | -- | $1,498,604,986 | $1,768,795,059 | 54.1 |
| All hospitalizations during the study period | $8,725,378,623 | $2,546,314,946 | $3,753,372,402 | $2,425,691,274 | 27.8 |

^a^High-cost users are defined as those in the top 10% of acute care costs based on prior years use. Non-high users are those in the bottom 90% of acute care costs who had at least one acute care admission in the prior 12 months.

^b^Terminal hospitalization is the hospital admission in which a person dies. The proximity of this hospitalization to death is reported in Table 2.

^c^Between high and non-high users in the 12 months prior to terminal hospitalization.

**eTable 3 – Procedural codes used to capture major surgery.**^2^

| **Anatomical Location** | **Procedural Codes** |
| --- | --- |
|  |  |
| Abdominal | A total of 703 CCI and CCP procedural codes were specified. Examples include gastrectomy and cholecystectomy. |
| Cardiac | A total of 737 CCI and CCP procedural codes were specified. Examples include coronary artery bypass grafting, valvular replacement, and heart transplant. |
| Retroperitoneal | A total of 287 CCI and CCP procedural codes were specified. Examples include surgeries involving bladder, ureter, and kidney. |
| Thoracic | A total of 465 CCI and CCP procedural codes were specified. Examples include pneumonectomy, wedge resection, and mediastinoscopy. |
| Vascular | A total of 853 CCI and CCP procedural codes were specified. Examples include aortic aneurysm repair, bypass grafting of peripheral arteries, and venous ligation. |
|  |  |

**eFigure 1 – Empirical cumulative distribution function (ECDF) curve of acute care costs in patients who died in hospital by timing of hospitalization in Canada between 2011 and 2015. Average costs of hospitalizations occurring in the 12 months prior to the terminal hospitalization (black line); Average costs of terminal hospitalization (red line). The “terminal hospitalization” is the hospitalization in which the patient died.**

**eReferences**

1. Juurlink DN, Preyra C, Croxford R, et al. *Canadian Institute for Health Information Discharge Abstract Database: a Validation Study*. Toronto; 2006.

2. Siddiqui NF, Coca SG, Devereaux PJ, et al. Secular trends in acute dialysis after elective major surgery--1995 to 2009. *CMAJ*. 2012;184(11):1237-1245. doi:10.1503/cmaj.110895.
